# The development and evaluation of a medical imaging training immersive environment

**DOI:** 10.1002/jmrs.60

**Published:** 2014-06-30

**Authors:** Pete Bridge, Therese Gunn, Lazaros Kastanis, Darren Pack, Pamela Rowntree, Debbie Starkey, Gaynor Mahoney, Clare Berry, Vicki Braithwaite, Kelly Wilson-Stewart

**Affiliations:** 1 School of Clinical Sciences, Queensland University of TechnologyBrisbane, Australia; 2 End-to-End VisualsBrisbane, Australia

**Keywords:** Evaluation, medical imaging, simulation, virtual reality

## Abstract

**Introduction:**

A novel realistic 3D virtual reality (VR) application has been developed to allow medical imaging students at Queensland University of Technology to practice radiographic techniques independently outside the usual radiography laboratory.

**Methods:**

A flexible agile development methodology was used to create the software rapidly and effectively. A 3D gaming environment and realistic models were used to engender presence in the software while tutor-determined gold standards enabled students to compare their performance and learn in a problem-based learning pedagogy.

**Results:**

Students reported high levels of satisfaction and perceived value and the software enabled up to 40 concurrent users to prepare for clinical practice. Student feedback also indicated that they found 3D to be of limited value in the desktop version compared to the usual 2D approach. A randomised comparison between groups receiving software-based and traditional practice measured performance in a formative role play with real equipment. The results of this work indicated superior performance with the equipment for the VR trained students (*P* = 0.0366) and confirmed the value of VR for enhancing 3D equipment-based problem-solving skills.

**Conclusions:**

Students practising projection techniques virtually performed better at role play assessments than students practising in a traditional radiography laboratory only. The application particularly helped with 3D equipment configuration, suggesting that teaching 3D problem solving is an ideal use of such medical equipment simulators. Ongoing development work aims to establish the role of VR software in preparing students for clinical practice with a range of medical imaging equipment.

## Introduction

There are many challenges facing clinical education for medical imaging students. The optimal situation would enable each student to practice a wide range of techniques on relevant equipment and with a diverse range of patients in a clinical department. In reality, this is affected by resource pressure faced by clinical placements and the associated quality of experience that the student will be able to benefit from. One of the aims of the 3-year Bachelor of Medical Imaging Science program at Queensland University of Technology (QUT) has been to provide students with the relevant underpinning knowledge, understanding and clinical skills to learn effectively in the clinical environment. Historically a key element of this, as in other institutions, has been skill development and practice with radiographic equipment and anthropomorphic models in an academic laboratory setting. With increasing cohort sizes this experience can present a considerable challenge to timetabling and resource availability. Access, maintenance, cost and risk of obsolescence are significant issues that can potentially be solved by using virtual reality (VR) simulation.

Educational environments that allow students to experiment with virtual equipment are widely used in a variety of clinical training scenarios and across a range of disciplines. Historically this development has largely been driven by medical training and the myriad of highly complex procedures that cannot allow trial and error. Opportunities for practice are severely restricted and traditionally have been limited to ‘in at the deep end’ experiences on patients who desperately need the procedure to be performed correctly. These scenarios are ideally suited to the use of VR simulators. VR applications have been successfully used to enhance training in procedures such as endotracheal tube placement,^1^ arthroscopy,^2^ bronchoscopy,^3^ IV catheter placement^4^ and laparoscopy.^5^ These simulations all provide training with small-scale equipment; however, radiographic equipment demands simulation of a ‘whole-room’ approach such as that employed in radiotherapy by the ‘Virtual Environment for Radiotherapy Training’ (VERT) software.^6^ Existing medical imaging simulation software solutions generally lack realism and the high degree of interactivity required for a true sense of presence to be engendered. Medical imaging students at QUT have been exposed to VERT in their first year as part of inter-professional learning activities undertaken with radiation therapy students. Informal feedback from these activities suggested strongly that medical imaging students could see the value of realistic VR simulation for their pre-clinical skills training. The published benefit of immersive visualisation for radiotherapy pre-clinical skills development^7^ also indicates that a realistic and flexible medical imaging immersive visualisation environment would be a highly valuable learning and teaching resource.

**Figure 1:**
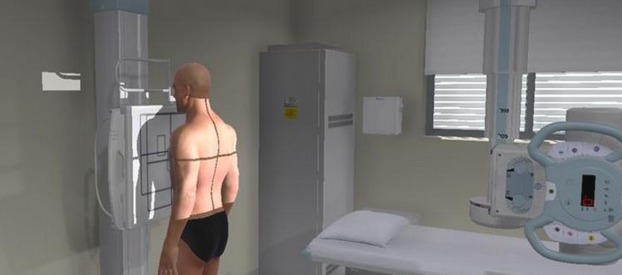
Screenshot of MITIE showing room, patient, tube and bucky stand. MITIE, medical imaging training immersive environment.

A novel simulated medical imaging training immersive environment (MITIE) was designed to provide medical imaging students with similar technical skills practice opportunities as their radiation therapy counterparts. Funding for this project was provided by Health Workforce Australia. Although there are VR radiography applications on the market it was hoped that a realistic and fully flexible 3D immersive environment could be developed to more accurately mimic clinical practice. From an educational perspective, it was felt important that the software should allow for experimentation while providing automated feedback including playback of procedures to highlight errors and potential improvements. Control systems were proposed to mimic clinical practice while allowing students to use the system without constant supervision. The proposed application comprised accurate 3D model of a digital radiography room, equipment and control area as seen in Figure 1. It was felt to be important that the software enabled students to image any patient position thus a fully adjustable and responsive patient model was constructed around a skeletal model. Images are generated directly from the skeletal model for optimal geometric accuracy. The software is inherently flexible with teaching staff able to create an unlimited range of their own ‘gold standard’ images and patient positions for students to compare.

The evaluation phase that this paper reports aimed to answer the following research questions:

- Is MITIE a potentially useful training tool for pre-clinical radiographic technical skills?
- Do students perceive MITIE to be of value for improving clinical skills?
- Do students find MITIE to be enjoyable and easy to use?
- Do students perceive a difference in usefulness of MITIE 3D compared to 2D?

## Methods

The MITIE simulation environment was created by End-to-End Visuals using the Quest3D runtime library. Quest3D is an advanced real-time development platform that leverages the Microsoft DirectX API. Quest3D was chosen for this application due to its high flexibility, rapid development environment and programmability. Custom rendering shaders were developed for the MITIE application along with a flexible animation rig for patient positioning. The MITIE application was developed in what is essentially a game development environment and as such requires a modern DirectX capable graphics card to perform efficiently. All rendering operations and calculations are performed on the graphics card with limited processes relying on the computer CPU. This affords a high-quality rendered environment with no discernible latency in operation. The software was capable of providing a standard 2D view or via 3D shutter glasses the environment could be interacted with in 3D. The MITIE simulation environment was developed using an agile development methodology according to the conceptual framework as outlined by Dingsøyr et al.^8^ The agile process utilised during the MITIE development was a highly iterative one with a large number of releases testing new features as they were added or modified. At peak development the project experienced up to two releases a week. This iterative process was driven by extensive consultation with a range of users resulting in minimal development time while addressing the needs of the project specifications. Users were given access to an online project monitoring interface that allowed them to submit bugs and add suggestions for improvements and enhancements. The strong collaborative and multi-disciplinary approach with extensive user consultation was directly responsible for the short time frame for development and release of the application.

During the development process a pilot study gathered user feedback from a focus group drawn from Year 3 (final year) medical imaging students. Students were provided with an overview of the software along with an opportunity to experiment with it individually. The evaluation process elicited student responses relating to use, enjoyment and value (perceived benefit) of the software. Users were asked to provide their thoughts regarding optimal and future use to inform ongoing development as part of the agile development process. Thematic analysis was performed in order to determine themes and address the evaluation aims.

User feedback was harvested over a period of 1 month from Year 2 medical imaging students after deployment of the final version. At this stage in their training these students had previously undertaken 5 weeks of clinical practice and a total of 33 h medical imaging-specific clinical skills laboratory training. The evaluation strategy comprised qualitative approach with Likert-style questionnaires collecting user demographic data, as well as responses concerning use of MITIE, satisfaction and perceptions of 3D use. Previous work^6^ has demonstrated poor correlation of VR training performance or enjoyment with age or gender thus although a good balance of demographics was seen in all arms of the studies these were not investigated in terms of factors affecting outcome. Open comment questions harvested student suggestions for optimal and future use for triangulation with Year 3 focus group responses.

All Year 2 Medical Imaging students were invited via email to participate in a study comparing MITIE and practical training. All 48 students consented to study involvement and were randomised into two cohorts, both receiving 2 h of training. One cohort received practical skills training for a specific radiographic technique using traditional X-ray clinical skills laboratory equipment and with the other cohort using just MITIE for the same specific radiographic technique. Both groups undertook a role play formative assessment where they were scored on their ability to ‘set up’ a volunteer for a designated radiographic projection. The evaluation specifically focussed on the students' ability to perform the projection. Since the data was not normally distributed, performance of students in the two cohorts was compared for statistical significance using the non-parametric two-tailed Mann–Whitney test in SPSS (SPSS Inc., Chicago, IL).

The study gained approval from QUT's University Human Research Ethics Committee (approval number 1300000005). Participation in the study was voluntary, although the teaching opportunities and formative assessment were available to all students irrespective of participation. Participants were informed that their decision to participate had no implications on their academic performance or development. Informed consent was gained from participants and anonymity of results was ensured throughout. All assessments were formative in nature and students who had not received MITIE or traditional training were provided with an opportunity to do so in order to guarantee equity of resource provision. No volunteers were irradiated; the assessment solely tested students' ability to arrange the ‘patient’ and equipment in the optimum configuration. Assessment of the students' performance was conducted by independent assessors who were blinded to the students' training method.

## Results

The three studies informing the evaluation aims were drawn from two different cohorts of students. Table 1 summarises cohort details along with users' self-perception of gaming experience (measured by time spent gaming).

**Table 1:** User demographic data (self-reported).

Evaluation	Year	Numbers	Mean MITIE hours per user	Gaming experience above average
Focus group	3	10	1.5	–
User satisfaction	2	50	3.8	54.5%
Performance test	2	48	2	58.5%

MITIE, medical imaging training immersive environment.

### Student satisfaction

Year 3 focus group responses related to the penultimate iteration of the software before release and their comments were fed into the final iteration. Students were able to outline the factors affecting their enjoyment and potential value of the software along with rationale for these. Overall responses were positive with a commonly expressed desire to commence use of the resource immediately and regret that they had not experienced it earlier in the course. Detailed discussions of themes arising are explored in the Discussion section.

Year 2 student satisfaction survey responses related to user experience of the final iteration, although specific issues arising during use were addressed promptly by the software development team. Quantitative results are presented in Table 2; there was considerable ambivalence illustrated by 30% of the response statements expressing neither opinion. The results did yield some trends, however; it can be seen that only 20% of students did not enjoy using MITIE and only 18% considered it to have been of no help in developing understanding of radiography techniques. Only 8% of students wanted more time with MITIE. The results also clearly showed that most students found the 3D version to be of less value than the 2D; this is explored in more detail in the Discussion section. Themes arising from the open comment questions from these students mostly related to specific issues with elements of the software which were consequently addressed by the development team. Included with these were some underlying issues about optimal use and these have been triangulated with those from the focus group discussion findings for further exploration in the Discussion section.

**Table 2:** User satisfaction results.

Likert statements	SD (%)	*D* (%)	*N* (%)	*A* (%)	SA (%)
I consider myself to be experienced with computers and/or gaming	2	20	16	44	18
I enjoyed using MITIE	2	18	46	30	4
It took me a long time to learn how to use MITIE	2	56	14	26	2
Overall I found MITIE easy to use	2	28	28	38	4
MITIE helped develop my understanding of general radiography techniques	2	16	36	38	8
I would have liked to have had more time using MITIE	14	40	38	6	2
‘3D’ MITIE was of more value for my learning than ‘2D’	32	34	32	2	0

Total respondents *n* = 50. SD, strongly disagree; D, disagree; N, neither; A, agree; SD, strongly disagree; MITIE, medical imaging training immersive environment.

### Performance evaluation

Performance evaluation was undertaken across the two cohorts through evaluation of student performance in a formative role play assessment activity. Results from students failing to select the correct projection were excluded from the evaluation as this learning was not an outcome of the training session. There was a small difference between the groups with 61% of MITIE students and 55% of the control group choosing the correct image technique. Complete evaluation forms were received from 22 MITIE group and 26 control group students. These students were scored using a scale from 1 to 5 on their ability to position the patient (‘patient position’ score), position the equipment (‘equipment position’ score) and their overall time taken (‘efficiency’ score) in their role play as summarised in Table 3. It can be seen that overall the MITIE group performed better than the control group with 91% of the MITIE students receiving an overall score above average (>3). A comparison of mean performance demonstrated statistical significance between the two groups.

**Table 3:** Performance scoring results.

Criterion	MITIE (*n* = 22)	Control (*n* = 26)	*P* value
Mean ‘patient position’ score	4	3.75	0.2501
‘Patient position’ score above average	91%	69%	–
Mean ‘equipment position’ score	4.18	3.62	0.0455
‘Equipment position’ score above average	77%	58%	–
Mean ‘efficiency’ score	4.23	3.69	0.1868
‘Efficiency score’ above average	77%	58%	–
Overall mean score	12.41	11.06	0.0366
‘Overall score’ above average	91%	77%	–

MITIE, medical imaging training immersive environment.

## Discussion

### Clinical training value

Most VR medical simulation applications share a common set of advantages over equipment-based simulation including cost efficiency for large numbers of students, a safe environment free from radiation or patient risks^9^ and reduced impact on laboratory staff or clinical workloads.^6^ MITIE has demonstrated similar advantages with up to 40 students able to concurrently practice techniques compared to 16 in the radiography laboratory under the supervision of two staff. The aim of this preliminary evaluation was to determine whether these advantages also extend to the effectiveness of VR-based skills practice when compared to equipment-based simulation. This could potentially pave the way for improved pre-clinical skills development and a possible reduction in clinical training hours as suggested by evidence from other professions.^5^,^10^ The results of this study indicate that MITIE has not only provided students with a more cost-effective and safe opportunity for practice but that it can also improve student performance with a simple radiographic technique role play assessment. It is interesting to note that although the overall score was improved for the MITIE group the individual element scores indicate that ability to arrange the equipment was the major influence on this score. Further study is in place to determine the extent to which this can be extended to enhance student performance in both academic and clinical skills assessment.

### Student enjoyment

Students clearly enjoyed using the software and the findings from the focus group and student surveys corroborate evidence in the literature.^6^ Students highlighted the similarity of the software to a game; one comment labelled it as ‘Perfect for our generation’. Feedback also indicated the value of resource availability outside demonstration times as a key factor. Since both enjoyment and support for independent learning were target aims of the software these were positive findings.

### Perceived benefits

Clearly independent learning should be fun and any improvement in student engagement has inherent value educationally. As well as enjoyment, however, students need to identify the value of the resource in order to engage fully. The findings of this study support the hypothesis that MITIE is useful for practising radiography techniques (with only 18% disagreeing). Themes from the qualitative data analysis triangulate well with this with students from both year groups identifying the value of MITIE for learning processes and techniques.

‘It was difficult to understand skull techniques from lectures but after using MITIE I began to grasp the different techniques’

(Student 1)

‘projections practised in MITIE were easier in labs’

(Student 2)

Some students did not share this enthusiasm with many comments emphasising how role play and practice with real people was of more value.

‘I would find it more useful and beneficial doing positioning on real people’

(Student 3)

This limitation was recognised from the outset by the development team but it is reassuring to discover that students acknowledge the importance of interpersonal skills. Other commonly perceived educational benefits of the software included the provision of ‘gold standards’ showing the correct positioning technique for comparison and provision of instant feedback. Automated feedback was a key factor in the success of the VERT evaluation

### Ease of use

There was ambivalence about the ease of use of MITIE and this may reflect the novelty of the resource during the initial piloting stage as the software was still being ‘tweaked’. Both year groups had plenty of comments relating to transient development bugs, suggestions for improved controls and shortcut keys. All of these suggestions were passed to the software development team and used to improve the application. There were comments relating to how MITIE might be better for ‘more tech savvy people’. The quantitative data contradicted this supposition, however, with no apparent influence of gaming experience on student perceptions.

### Benefit of 3D

Student feedback indicates that they found the 3D mode of the software to be less useful than the 2D; most students used the 2D mode exclusively. This could have been influenced by teething problems with the 3D hardware or by side effects of 3D use. Comments from two students certainly suggested that use of 3D had led to headaches, although Flinton and White^11^ found no evidence of this with VERT. It is more likely that the reported preference for 2D is related to the desktop screen size. Research suggests that 3D visualisation is useful in situations where spatial awareness is required and since medical imaging technique is essentially a 3D problem it would seem logical that 3D would be of value in this situation. The findings of the study strongly contradict this supposition with only one student agreeing. A 2010 study^12^ noted that immersion requires a large field of view and this suggests that the desktop nature of the software may reduce the impact of the 3D environment. Further evidence is required in order to clarify the value and potential role of 3D visualisation within medical imaging training. A balance must be attained between large training numbers (based on desktops) and level of immersion (based on resource-intensive large-screen equipment).

### Software limitations

As with other VR simulations, students cited the limitations of the software as being related to the lack of patient interaction (both verbal and tactile) and it is clear that the role of MITIE needs to be strongly limited to pre-clinical technical skills training. It is not a replacement for clinical practice but does purport to help students concentrate on patient-related skills rather than technical issues thus optimising placement time.

### Pedagogical implications

Unsupervised practice with X-ray equipment is impossible due to health and safety, radiation protection and supervision requirements; this is not the case for VR-based simulation. Apart from the initial teaching session where students were introduced to the software, a staff:student ratio of 40:1 is feasible with MITIE and highly efficient. Inherent in the reduction in supervision and increased efficiency of resource use is the increase in time available for practice per student. Students are able to practice a range of routine procedures with minimal supervision and automated feedback to ensure they are familiar with basic techniques and control systems before attending laboratory practice or clinical placements. A major value of the application is that students are able to experiment and see resultant images of incorrect setups. Unlike in other professions where the purpose of simulation is to practice procedures to minimise error; in medical imaging there is great value in being able to visualise the effect of incorrect technique as this reinforces the importance of accurate positioning and allows reflection on corrective approaches. Thus, supervised teaching in X-ray laboratories can be readily supplemented with independent experimentation and student-directed MITIE practice. This enables supervised teaching sessions to reduce the emphasis on positioning of equipment and focus on establishing patient interaction skills and applying essential underpinning knowledge to the finer points of techniques and image recognition. Building on the results of this study MITIE is now being embedded across the curriculum to provide a resource that can build skills in a continuous fashion and complement the existing laboratory training. Looking to the future, the rapid rate of technological development means that ensuring the latest equipment is available for students to practice with in an academic environment is prohibitively expensive. Software models are relatively easy and inexpensive to update and MITIE purports to help ensure medical imaging education at QUT remains at the cutting edge of current developments.

### Study limitations

The limitations of the evaluation were that user responses were drawn during software development and thus may be coloured by issues that were subsequently addressed as part of the agile development process. Further evaluation of the most recent software version is currently ongoing. Although focus group recruitment and survey response rates were adequate, the performance testing numbers were severely reduced when students chose the incorrect projection. It should also be noted that this study only aimed to determine whether MITIE could be used for medical imaging technique training based on a single projection and further work is ongoing to determine the pedagogical link between more widespread MITIE exposure and clinical skills.

### Future potential directions

Additional CT and C-arm fluoroscopy modules have also been developed to allow students to train with different modalities; evaluation of these resources is ongoing. The software architecture could also be developed in the near future to provide resources for other modalities including MRI and PET. The environment makes an ideal tool for inter-professional education and can provide an efficient and realistic overview of different roles and procedures. Due to the visual appeal of such applications it is also likely to play an important role in recruitment of suitable students. Student feedback comments requested the ability to communicate and touch the patient and potential inclusion of haptic devices^13^ and avatar technology^14^ could enable this. It is uncertain as to whether the perceived value of these innovations would add significant educational value to the application to warrant the considerable expense and reduction in large-scale use.

## Conclusions

A realistic medical imaging VR software application has been developed using a gaming environment, accurate modelling and a high level of interactivity. The agile development methodology used led to a fast and flexible process. The MITIE software enables large group practice of techniques in a safe environment and more efficient use of radiographic laboratory time. Students practising projection techniques with MITIE performed better at role play assessments than students practising in a traditional radiography laboratory only. The VR application in this case helped students in particular to arrange the equipment in the correct 3D configuration, suggesting that teaching 3D problem solving is an ideal use of such medical equipment simulators. Students enjoyed using the software and perceived real educational value from the independent learning and process learning potential. There was no perceived benefit associated with desktop 3D for the application and no clear impact of gaming experience on student perceptions. Further research into the potential role of large-screen 3D immersion as well as MITIE's impact on clinical placement performance is ongoing.

## Supporting Information

**Video S1.** A screencast of the medical imaging training immersive environment (MITIE) being used to practice a simple radiograph of the hand. This video was taken using the 2D mode of the software. Source: Queensland University of Technology, Australia.
